# The anti-allergic activity of *Cymbopogon citratus* is mediated via inhibition of nuclear factor kappa B (Nf-Κb) activation

**DOI:** 10.1186/s12906-015-0702-8

**Published:** 2015-06-06

**Authors:** Marta Santos Serafim Machado, Hugo Bernardino Ferreira Silva, Raimon Rios, Anaque Pires de Oliveira, Noma Vilany Queiroz Carneiro, Ryan Santos Costa, William Santos Alves, Fabio-Luis Meneses Souza, Eudes da Silva Velozo, Silvana Alves de Souza, Tania Maria Sarmento Silva, Maria Lenise Silva, Lain Carlos Pontes-de-Carvalho, Neuza Maria Alcântara-Neves, Camila Alexandrina Figueiredo

**Affiliations:** Departamento de Biorregulação, Instituto de Ciências da Saúde, Universidade Federal da Bahia, Campus do Canela, CEP 41110-100 Salvador, BA Brazil; Centro de pesquisa Gonçalo Moniz, Fundação Oswaldo Cruz, Salvador, Bahia Brazil; Faculdade Adventista da Bahia, Cachoeira, Bahia Brazil; Faculdade de Farmácia da Universidade Federal da Bahia, Salvador, Bahia Brazil; Departamento de Ciências Moleculares, Laboratório de Bioprospecção Fitoquímica da Universidade Federal Rural de Pernambuco, 52171-900 Recife, Pernambuco Brazil; Instituto de Biologia, Universidade Federal da Bahia, Salvador, Bahia Brazil

**Keywords:** *Cymbopogon citratus*, Asthma, *Blomia tropicalis*, Natural products

## Abstract

**Background:**

The prevalence of allergic diseases such as asthma has significantly increased worldwide, making it a public health concern. There is an urgent need for new anti-inflammatory agents with selective pharmacology and lower toxicity. Plant extracts have been used for centuries in traditional medicine to alleviate inflammatory diseases. In this work, we evaluated the anti-allergic activity of *Cymbopogon citratus* (*Cy)*, a medicinal herb used by folk medicine to treat asthma.

**Methods:**

We used a murine model of respiratory allergy to the mite *Blomia tropicalis* (*Bt*) and evaluated certain parameters known to be altered in this model. A/J mice were sensitized (100 μg/animal s.c.) and challenged (10 μg/animal i.n.) with *Bt* mite extract and treated with 60, 120 or 180 mg/kg of *Cy* standardized hexane extract. The parameters evaluated included: cellular infiltrate in bronchoalveolar lavage (BAL); eosinophil peroxidase activity (EPO); histopathological examination of the lung; serum levels of specific IgE, IgG1 and IgG2a; Th2 cytokine concentrations in BAL and expression of NF-κB.

**Results:**

Our results showed that oral administration of a *Cy* hexane extract (especially 180 mg/Kg) reduced the numbers of leukocytes/eosinophils in BAL; the eosinophil peroxidase activity in BAL; the infiltration of leukocytes in lung tissue; the production of mucus in the respiratory tract; the level of IL-4 in BAL and the nuclear expression of NF-κB.

**Conclusions:**

The results presented demonstrate the potential of the *Cy* hexane extract to modulate allergic asthma; this extract may be an alternative future approach to treat this pathology.

## Background

The prevalence of allergic diseases such as asthma has increased significantly over the past few years in developed and developing countries [1]. Dust mites are the main sources of airborne allergens, and *Blomia tropicalis* is an important sensitizing agent in tropical regions, contributing to the asthma prevalence in Brazil [2]. Allergic bronchial asthma is a complex syndrome characterized by airflow obstruction, bronchial hyper-responsiveness and airway inflammation [3] driven by T-helper type 2 (Th2) cytokines, including IL-4, IL-5 and IL-13 [4]. These cytokines promote IgE synthesis, stimulate eosinophil growth and differentiation, and increase mucus production [5]. Although the critical roles of Th2 cytokines in the pathogenesis of allergic asthma have been established, the mechanisms for overproduction of Th2 cytokines in asthmatic responses are not fully understood [6]. Current guidelines for asthma treatment recommend first-line anti-inflammatory therapy with inhaled and, in the most severe cases, systemic corticosteroids that are effective at controlling allergic airway inflammation in most cases; however, they also pose a significant risk for adverse effects, such as general or local immune suppression, diabetes, and adiposity [7]. New or complementary anti-inflammatory drugs for asthma therapy are necessary. Historically, herbal medicine has been very important in the treatment of asthma. Plant extracts and their components are known to exhibit biological activities [8]. Our research group has standardized a model of allergy using the mite *Bt* [9], obtaining therapeutic results with *Cissampelos sympodialis* and its alkaloid warifteine [10], as well as *Ocimum gratissimum* methanolic extract and rosmarinic acid [11], in the control of allergic asthma. Our objective in the present study was to investigate the immunomodulatory effects of *Cymbopogon citratus(Cy)* and its possible mechanisms in a murine model of allergy to *Blomia tropicalis*. The species (Poaceae-Gramineae), commonly known as lemongrass, is a spontaneous perennial grass that is largely distributed around the world, especially in tropical and subtropical countries [12]. Our group’s interest in investigating this plant was because it was often used as a treatment of asthma and other respiratory allergies by folk medicine in the city of Salvador, Bahia, Brazil [13]. Extracts of dried *Cy* leaves are used in traditional medicine for the treatment of inflammation, digestive disorders, diabetes, nervous disorders, and fever, as well as other health problems [14]. No report has been found referring to the anti-allergic property of this species. However, there is evidence of an anti-inflammatory property, especially the ability of the essential oils from *Cy* to inhibit iNOS and NF-κB [14]. Understanding the molecular mechanisms underlying the healing properties of natural products is crucial to finding compounds that could be useful as templates for new therapeutic drugs in the treatment of allergic diseases.

## Methods

### Animals

Male A/J mice (25–30 g) were used throughout the study. Animals were maintained with free access to food and water. They were obtained from the animal facilities of the Fundação Oswaldo Cruz, Bahia, Brazil. Groups of five animals were used in each experiment. All experimental procedures were approved by the Ethical Committee for Use of Experimental Animals of the Faculdade de Odontologia, Universidade Federal da Bahia, Brazil (protocol number: 02/09), and conducted according to international standards.

### *Blomia tropicalis* extract

*Blomia tropicalis* mites were cultivated in a fish food-containing standardized environment, purified with saturated NaCl and lysed in a blender (51BL30; Waring Commercial, Torrington, CO, USA) in 0.15 M phosphate-buffered saline, pH 7.4 (PBS). After several centrifugations with ether (9000 × g for 10 min) to remove lipids, protein content was determined by Lowry’s method [15]; the extract was stored at −20 °C until use. *Bt* extract was standardized by determining the Blo t 5 allergen concentration using a commercial capture ELISA (INDOOR Biotechnologies, Charlottesville, VA, USA). All batches of *Bt* that were used contained 30–40 ng allergen per μg of protein.

### Preparation of hexane extract from *Cymbopogon citratus*

Leaves of *Cymbopogon citratus* were obtained from Cachoeira, Bahia, Brazil and a voucher specimen was deposited in the herbarium from the Institute of Biology, Federal University of Bahia (UFBA), ALCB 98522. Preparation of the *Cy* hexane extract was performed according to the technique described by Shetty et al. (2008) in the research laboratory in the medical field (LAPEMM) of the UFBA [16]. Briefly, dry plant material was pulverized, and crude extract was prepared by a successive maceration process using hexane. After filtration, the extracts were concentrated under vacuum at 40 °C.

### HPLC-DAD-ESI-MS and CG-MS

HPLC analysis was performed in a Shimadzu Prominence LC-20AT equipped with a SPD-M20A diode array detector (Shimadzu Corp. Kyoto, Japan). The samples were injected into a Rheodyne 7125i injector with a 20 μl loop. The column heater was set at 40 °C. The chromatographic separation was performed with a Luna Phenomenex C-18 column (250 mm × 4.6 mm × 5 μm). The compounds were separated using a mobile phase consisting of 5 % aqueous formic acid (A) and methanol (B) at a flow rate of 1 mL/min. The separation gradient was 0–20 min 50–70 % B, 20–40 min 70–90 % B, 40–50 min 90–100 % B, 50–60 min 100 % B. The injection volume was 20 μL. Chromatograms were recorded at 320 nm. The HPLC-MS was obtained in positive electrospray mode using an MS (Esquire 3000 Plus, Bruker Daltonics, Germany), capillary voltage 4500 V, nebulizer pressure 26 psi, drying gas 6.0 l/min, and gas temperature 325 °C.

Gas chromatography coupled with mass spectrometry was performed using a GC-MS-QP5050 (Shimadzu Corp. Kyoto, Japan), equipped with BPX5 (5 % phenyl polysilphenylene siloxane) a capillary column (30 m × 0*.*25 mm i.d.,0.25 μm). For GC-MS detection, an electron ionization system was used with ionization energy of 70 eV. Helium was the carrier gas, at a flow rate of 1.7 mL/min. Injector temperature 240 °C, detector temperature 230 °C, temperature program, temperature program: 50 °C (5 min)-250 °C, 5 °C/min. The identification of the components was made through comparison of sbstance mass spectrum with the database of the GC/MS (Willey229, NIST107, SHIM1607, NIST21).

The extraction of phenolics compounds from *Cymbopogon citratus* extract was performed using C18 cartridge. 100 mg of extract was suspended into 1 mL of MeOH and filtered and filtered to separate the precipitate. The C18 cartridge (SEP-PAK Waters) was sequentially conditioned with 10 mL of MeOH and 10 mL of acidified water (pH 2.0 with HCl) without allowing the cartridge to dry. The filtrate was passed through the cartridge and rinsed with 10 mL of water and the phenolic compounds were eluted with 10 mL of HPLC-grade methanol. The eluate was dried under Nitrogen gas furnished 18.2 mg of phenolic fraction. This fraction was dissolved in methanol, filtered through a 0.45-μm nylon syringe filter (Whatman) and injected into the HPLC system. The insoluble precipitate in methanol was solubilized with dichloromethane for GC-MS analysis.

### Cytotoxicity assay

The cytotoxicity of *Cy* was evaluated as described by Stevignyetal (2002) using the tetrazolium salt MTT (3–4,5-dimethylthiazol-2-yl)-2,5diphenyltetra-zolium bromide (Sigma) colorimetric method based on the cleavage of the reagent by dehydrogenases in viable cells (Mosmann, 1983). Stock solutions of the *Cy* hexane extract were prepared at 10 mg/mL in DMSO. The solutions were further diluted to final concentrations of 1000–1.95 μg/mL. Cultures were incubated for 48 h at 37 ° C in an atmosphere with 5 % CO_2_. All experiments were performed at least in duplicate.

### Sensitization and challenge with *Bt* antigen

The murine model of respiratory allergy was induced as previously described [17]. Briefly, A/J mice (*n* = 5) were initially sensitized by two subcutaneous injections (day 0 and day 7) of Bt (10 μg of protein) adsorbed to 4 mg/mL Al(OH)_3_ in saline. 24 hours after the last subcutaneous injection, animals received three intranasal immunization boosters/challenges with Bt (10 μg/instillation) every other day. Two days after the last immunization booster/challenge, they received a final intranasal challenge of 10 μg Bt. The negative control group received saline in both sensitization and challenge procedures. 24 h after the last challenge, the animals were euthanized with intraperitoneal injections of xylazine and ketamine (40 mg/kg/body weight).

### Treatment with *Cymbopogon citratus*

**Fig. 1:**
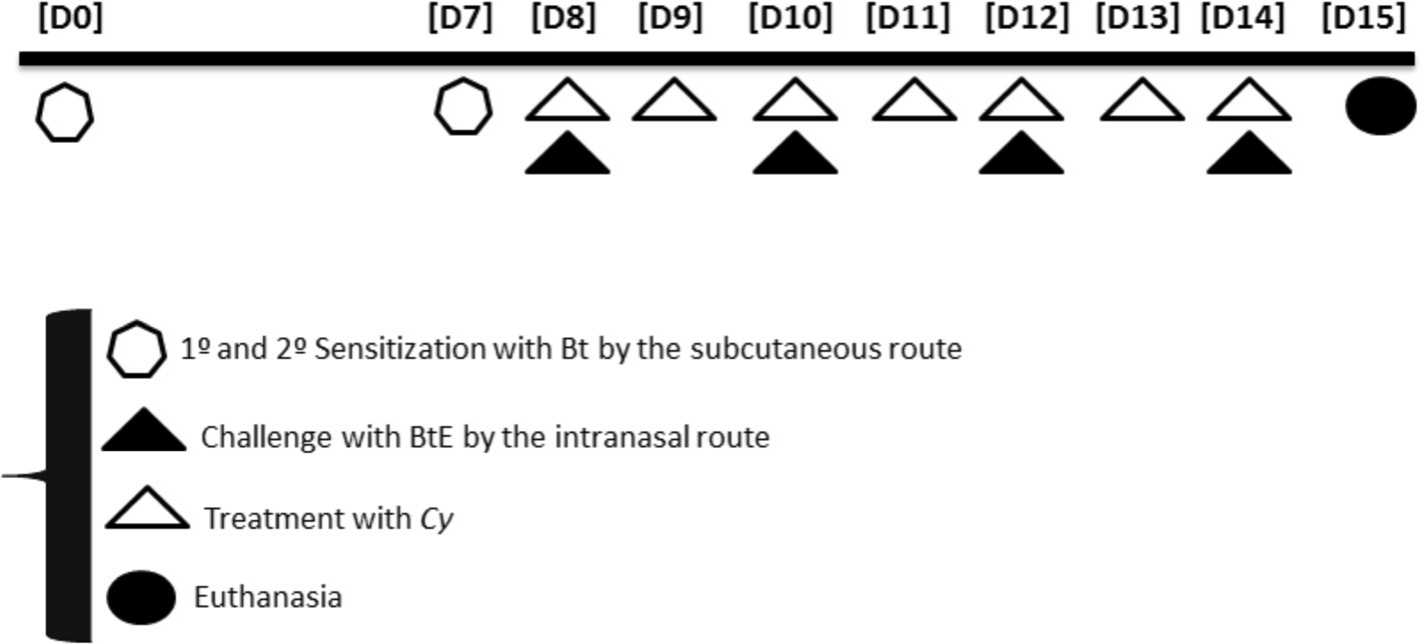
Experimental protocol for induction of respiratory allergy. Experimental protocol for induction of respiratory allergy using aluminum hydroxide-adsorbed *Blomia tropicalis* extract (BtE) and assessment of the treatment with *Cy* hexane extract (60, 120 or 180 mg/kg, orally). [D1] to [D15], days 1 to 15 of experiments

The different groups were treated daily from the 8th to the 14th day of the experimental protocol, one hour after intranasal challenges on the 8th, 10th, 12th, and 14th days (Fig. 1). Animals were orally administered 60, 120 or 180 mg/kg of *Cy* or 3 mg/kg of dexamethasone (Dex). The groups of animals were named: Control, non-sensitized and saline-treated mice; Bt, Bt-sensitized and saline-treated mice; Bt/CcH60, Bt/CcH120 and Bt/CcH180, Bt-sensitized, Bt-challenged and *Cy*-treated mice; Bt/Dex, Bt-sensitized and Dex-treated mice.

### Bronchoalveolar lavage (BAL)

The trachea was cannulated, and the lungs were carefully washed three times with 0.5 mL PBS containing 1 % bovine serum albumin (BSA). The total number of leukocytes in the BAL was immediately determined in a hemocytometer using Trypan blue. Differential cell counts were obtained using May–Grunwald–Giemsa-stained cytospin preparations. A differential count of at least 100 cells was performed in a blind fashion in accordance with standard morphologic criteria.

### Eosinophil peroxidase (EPO) activity

EPO activity in cells obtained from BAL was measured according to a previously described method [18]. Briefly, cell suspensions were frozen and thawed three times in liquid nitrogen. After centrifugation at 4 °C for 10 min at 1000 x g, cell lysates were placed into 96-well plates (75 μL/well), followed by the addition of 150 μL of the chromogen and substrate solution (1.5 mmol/L o-phenylenediamine and 6.6 mmol/L H_2_O_2_ in 0.05 mol/L Tris–HCl, pH 8.0). After 30 min at room temperature, the reaction was stopped by the addition of 75 μL of 0.2 mol/L citric acid; the absorbance of the samples was determined at 492 nm using an ELISA reader.

### Levels of IL-4 in bronchoalveolar lavage (BAL)

The concentrations of IL-4 in BAL were quantified by a standard ELISA as recommended by the manufacturer (BD Pharmingen, USA).

### Measurement of anti-Bt IgE, IgG1 and IgG2a antibody levels in serum

Anti-Bt antibody levels in serum from the different experimental groups were determined by indirect ELISA: 96-well micro titer high-binding plates (Costar, Cambridge, MA, USA) were coated with Bt (100 μg/well) overnight at 4 °C and blocked for 1 h with PBS-T containing 10 % fetal calf serum (FCS, Gibco, Pisley, UK) at room temperature (RT). After this incubation period, the serum samples were added, and the plates were incubated overnight at 4 °C. Biotin-conjugated IgE, IgG1 or IgG2a anti-mouse (BD Pharmingen, San Diego, CA, USA) was added to the wells and incubated for 1 h at RT. A solution of avidin-horseradish peroxidase (BD Pharmingen, San Diego, CA, USA) was then added to each well for 30 min. Finally, a solution containing 3,3′,5,5′-tetramethylbenzidine and hydrogen peroxide (BD Pharmingen, San Diego, CA, USA) was added and incubated for 30 min at RT; the reaction was then stopped with 4 M sulfuric acid. The absorbance of the sample was determined at 492 nm using an ELISA reader.

### Histopathological analysis

The degree of peribronchiolar and perivascular inflammation was evaluated as described previously [19]. Briefly, lung tissues were fixed by inflation with freshly prepared 10 % (v/v) paraformaldehyde. The specimens were dehydrated and embedded in paraffin. Tissue sections (5 μm) were stained with hematoxylin and eosin to assess cellular infiltration under optical microscopy with 400× magnification. The data on quantification of lung inflammation were acquired using the software Image-Pro Plus Version 6.1 (Media Cybernetics, San Diego, CA, USA) using the inflammatory area index. Additionally, tissue sections were stained with periodic acid-Schiff to assess the presence of mucus. A quantitative digital analysis was performed as described previously [9].

### Detection of NF-κB/p65 in lung tissue by Western blot

Lung tissue (200 mg) from each group of mice was homogenized with lysis buffer (1000 μl RIPA). The samples were centrifuged for 30 min at 200 × g and 4 °C, and the supernatant was collected. Protein concentration was determined by Lowry’s method [16]; 20 μg protein from each group was separated by 13 % SDS-PAGE (Bio Rad, Blot SD – MO – USA, 100 v 1.5 h) and electrotransferred to a nitrocellulose membrane (Hybound – ECL, Amersham Biosciences, Bio Rad, Blot SD – MO – USA, 15v 30 min). The membrane was blocked with 3 % BSA (3 g BSA was dissolved in 100 ml PBS) for 2 h at 37 °C and incubated with polyclonal rabbit anti-NF-kB (1:100) overnight at 4 °C. The membrane was washed with TBST (Tris–HCl pH7.5 5 ml, 20 % Tween-20 1.18 ml, NaCl 4.4 g, 500 ml dd Water) for 2 × 10 min, incubated with HRP labeled secondary monoclonal antibody (1:1000) for 1.0 h at 37 °C, washed with TBST 2 × 10 min and visualized using an ECL Western blot analysis system (GE Healthcare).

### Detection of NF-κB/p65 in the lung tissue by immunohistochemistry

Lung sections (4 μm) obtained from paraffin blocks were placed on glass slides treated with poly-L-lysine (Sigma Diagnostic). The exposure of antigenic epitopes was achieved by heating (97 ° C) in citrate buffer pH 6.0 for 30 min. Endogenous blocking was performed with 3 % hydrogen peroxide in methanol for 15 min and then block was performed for nonspecific binding with 4 mg/ml bovine serum albumin (BSA) for 20 min. NF-κB/p65 antibody (Sigma) was added to tissue at a concentration of 1:80 at 4 ° C for 12 h. After washing with PBST, the sections were incubated with the secondary antibody, biotinylated IgG (Sigma 1:1000), for 1 h at room temperature. Signal amplification was performed using streptavidin-peroxidase (Pearce 1:1000). After development of the slides with DAB 3,3′ diaminobenzidine (DAB, Dako, Carpinteria, USA), the slides were counter-stained with hematoxylin and mounted with glass coverslips containing Canada balsam (Riedel de Haen AG, Hannover, Germany). The data on quantification of NF-κB expression were acquired using the software Image-Pro Plus Version 6.1 (Media Cybernetics, San Diego, CA, USA) using the expression area index.

### Statistical analysis

One-way analysis of variance (ANOVA) and Tukey’s post-test (for data with normal distribution) were used to determine the significance between experimental groups. Differences with *p* values ≤ 0.05 were considered statistically significant. Each experiment was repeated at least two times.

## Results

### Standardization hexane extract from *Cymbopogon citratus*

**Fig. 2:**
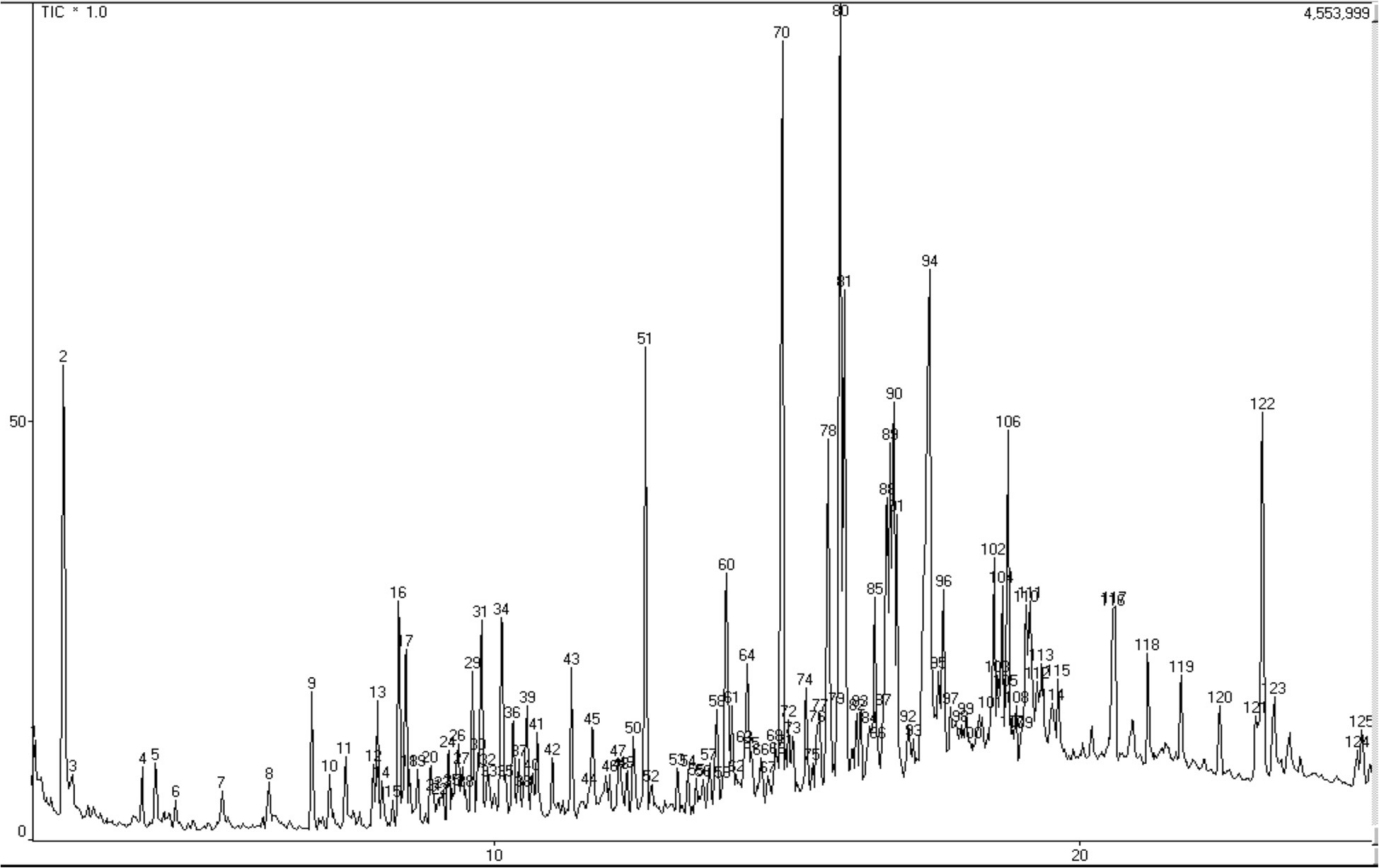
Chromatogram obtained by gas chromatography coupled with mass spectroscopy

**Fig. 3:**
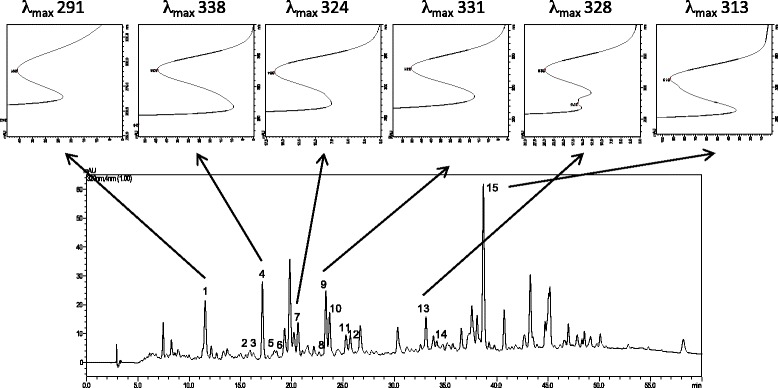
HPLC-DAD (320 nm) chromatogram of phenolic fraction of *Cy* and UV of representative compounds 1, 4, 7, 8, 13 and 15

**Fig. 4:**
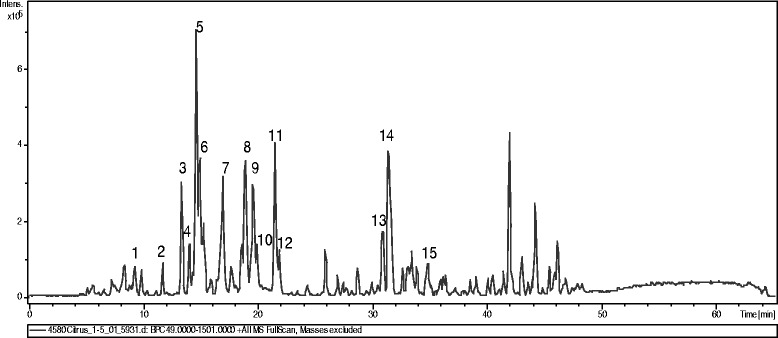
TIC chromatogram of phenolic fraction of *Cy* from HPLC (+) ESI-MS

The chromatogram obtained by gas chromatography coupled with mass spectrometry is presented in Fig. 2. The analysis showed the main compounds: 2-hydroxy-4-methyl2-pentanone (3.89 %), elemol (2.53 %), beta esdesmol (2.30 %), oplapanone (4.79 %), nerolidol epoxyacetate (2.78 %), phytol acetate (5.66 %), eicosanoic acid methyl ester (1.89 %), palmitic acid (8.49 %) and phytol (1.82). The phenolic composition of *Cy* extract was investigated using a HPLC method with diode array and mass spectrometric detection. 15 compounds tentative peak assignment partly were assigned as sinapic acid derivatives with the exception of substances four and five which can be derived from ferulic acid. The compounds derivatives of sinapic acid may be characterized as sinapate esters. The major fragments were found at *m/z* 221, 203 and 147. The fragments to esters of ferulic acid were observed at *m/z* 195, 155 and 137. The UV spectra (Figs. 3 and 4) corroborate the proposal for derivatives of cinnamic acids because the maximum lambda values observed between 290 and 338 nm nm [20].

### *Cymbopogon citratus* has no significant cytotoxicity in spleen cells from mice

**Fig. 5:**
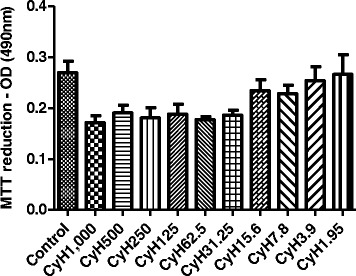
Evaluation of the cytotoxicity of *Cymbopogon citratus* hexane extract on cultured splenocytes of mice sensitized with *Bt* antigen. Effect of hexane extract of *Cymbopogon citratus* (CyH) on spleen cells. Cells were exposed to supplemented RPMI medium (Control) or supplemented RPMI medium and various concentrations of CyH (1000 at 1,95 μg/ml) by 48 h at 37 °C in an atmosphere with 5 % CO_2_. The MTT assay was performed as described in Materials and Methods

Our results show that cultures treated with different concentrations of *Cy* not had significant differences of dead cells*,* when compared to the negative control. Thus, *Cy* has no cytotoxicity at the concentrations evaluated (Fig. 5).

### Treatment with *Cymbopogon citratus* hexane extract decreases the number of inflammatory cells and eosinophil peroxidase (EPO) levels in mice sensitized with *Bt* antigen

**Fig. 6:**
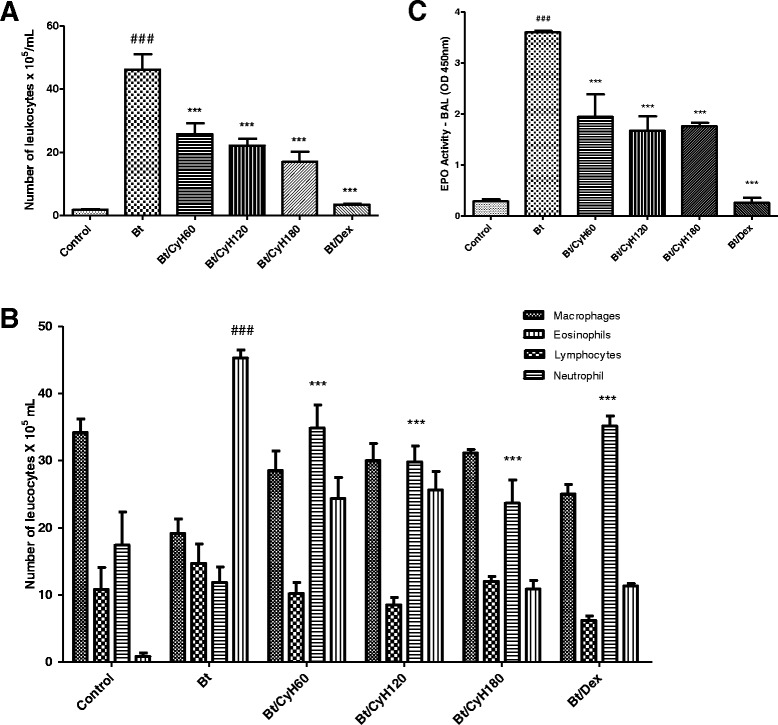
Effect of *Cymbopogon citratus* on leukocytes and EPO activity in the bronchoalveolar lavage (BAL) of Bt-sensitized and challenged A/J mice. **a** Total cells counted in BAL; **b** eosinophilia in BAL; **c** eosinophil peroxidase (EPO) activity in BAL. Animals were sensitized and treated with saline (*Control*), sensitized with *Blomia tropicalis* (100 μg/animal) and 4 mg/mL of [AL(OH)_3_] and treated with saline (Bt), sensitized with *Blomia tropicalis* (100 μg/animal) and 4 mg/mL of [AL(OH)_3_] and treated with *Cy* hexane extract (CyH) at 60 mg/kg (Bt/CyH60), 120 mg/kg (Bt/CyH120), 180 mg/kg (Bt/CyH180) or 3 mg/kg dexamethasone (Bt/Dex). Columns represent the mean values of the results obtained from five animals, and error bars represent the standard deviation from the means. ### *p* < 0.001 vs. control; ***p < 0.001 vs. Bt group by ANOVA-Tukey

As shown in Fig. 6, an increase in total leukocytes and eosinophils in BAL of Bt-sensitized animals was observed when compared to the negative control group (*p* < 0.001; Fig. 6a). Among the groups treated with *Cy* extract, daily oral administration of 60, 120 or 180 mg/kg resulted in a significant reduction in the number of inflammatory cells and eosinophils (*p* < 0.001; Fig. 6B) when compared to Bt-sensitized animals. Animals sensitized with *Bt* had higher EPO activity in lung tissue (*p* < 0.001; Fig. 6C) compared to the control group. Mice treated with *Cy* hexane extract showed a significant reduction in EPO levels in the BAL at all administered doses (*p* < 0.001; Fig. 6c). As expected, the oral administration of 3 mg/kg Dex significantly suppressed the number of eosinophils, total inflammatory cells and EPO levels (*p* < 0.001; Fig. 6c).

### Treatment with *Cymbopogon citratus* hexane extract decreases the levels of Bt-specific IgE, IgG1 and IgG2a in the serum of mice sensitized with *Bt* antigen

**Fig. 7:**
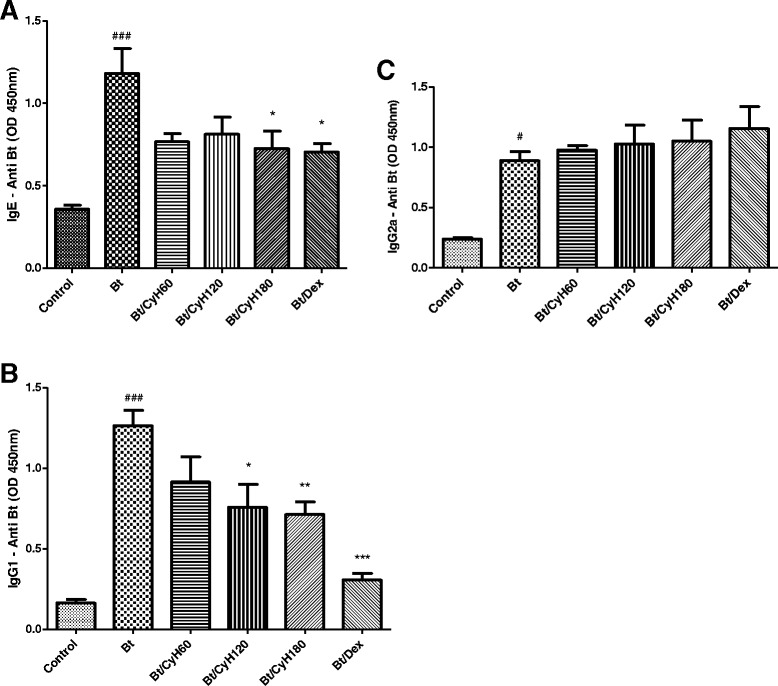
Effect of *Cymbopogon citratus* on the production of IgE (**a**), IgG1 (**b**) and IgG2a (**c**) in the serum of mice sensitized with *Bt* antigen. A/J mice were sensitized and treated with saline (*Control*), sensitized with *Blomia tropicalis* (100 μg/animal) and 4 mg/mL of [AL(OH)_3_] and treated with saline (Bt), sensitized with *Blomia tropicalis* (100 μg/animal) and 4 mg/mL of [AL(OH)_3_] and treated with *Cymbopogon citratus* hexane extract (*CyH*) at 60 mg/kg (Bt/CyH60), 120 mg/kg (Bt/CyH120), 180 mg/kg (Bt/CyH180) or 3 mg/kg dexamethasone (Bt/Dex). Columns represent the mean values of results obtained from five animals, and error bars represent the standard deviation from the means. ### *p* < 0.001 vs. control; *p < 0.05; **p < 0.01; ***p < 0.001 vs. Bt group by ANOVA-Tukey

Figure 7 presents the levels of anti-IgE, IgG1 and IgG2a specific for *Bt* in sensitized and treated animals. As shown in this figure, mite sensitization induced high levels of these antibodies when compared to the control group (*p* < 0.001; Fig. 7a-c). Treatment with 180 mg/kg of *Cy* significantly reduced the level of IgE (*p* < 0.01; Fig. 7a). The oral administration of 3 mg/kg Dex significantly reduced the level of IgE (*p* < 0.01; Fig. 7a).

Daily oral administration of 120 mg/kg (*p* < 0.05; Fig. 3b) or 180 (*p* < 0.01; Fig. 7b) reduced the levels of IgG1 in the serum of Bt-sensitized mice. Treatment with 3 mg/kg Dex decreased levels of IgG1 (*p* < 0.001; Fig. 7b) in Bt-immunized and challenged mice. Treatment with *Cy* did not significantly reduce IgG2a serum immunoglobulins levels (Fig. 7c).

### Treatment with *Cymbopogon citratus* hexane extract decreases IL-4 levels in the BAL of Bt-sensitized mice

**Fig. 8:**
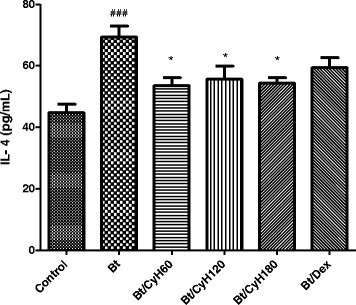
Effect of *Cymbopogon citratus* on the cytokine IL-4 in BAL of mice sensitized with Bt antigen. A/J mice were sensitized and treated with saline (*Control*), sensitized with *Blomia tropicalis* (100 μg/animal) and 4 mg/mL of [AL(OH)_3_] and treated with saline (Bt), sensitized with *Blomia tropicalis* (100 μg/animal) and 4 mg/mL of [AL(OH)_3_] and treated with *Cymbopogon citratus* hexane extract (CyH) at 60 mg/kg (Bt/CyH60), 120 mg/kg (Bt/CyH120), 180 mg/kg (Bt/CyH180) or 3 mg/kg dexamethasone (Bt/Dex). Columns represent the mean values of results obtained from five animals, and error bars represent the standard deviation from the means. ### *p* < 0.001 vs. control; *p < 0.05; **p < 0.01; ***p < 0.001 vs. Bt group by ANOVA-Tukey

Levels of IL-4 in the BAL were higher in Bt-immunized and challenged mice compared to the control group (*p* < 0.05; Fig. 8). Treatment with 60, 120 or 180 mg/kg *Cy* hexane extract led to significant reductions in the levels of IL-4 (*p* < 0.05; Fig. 8). No reduction was observed in dexamethasone- treated animals compared to mice sensitized with *Bt* and treated with saline (Fig. 8).

### Treatment with *Cymbopogon citratus* hexane extract decreases inflammatory cell infiltration in lung tissue of mice sensitized with *Bt* antigen

**Fig. 9:**
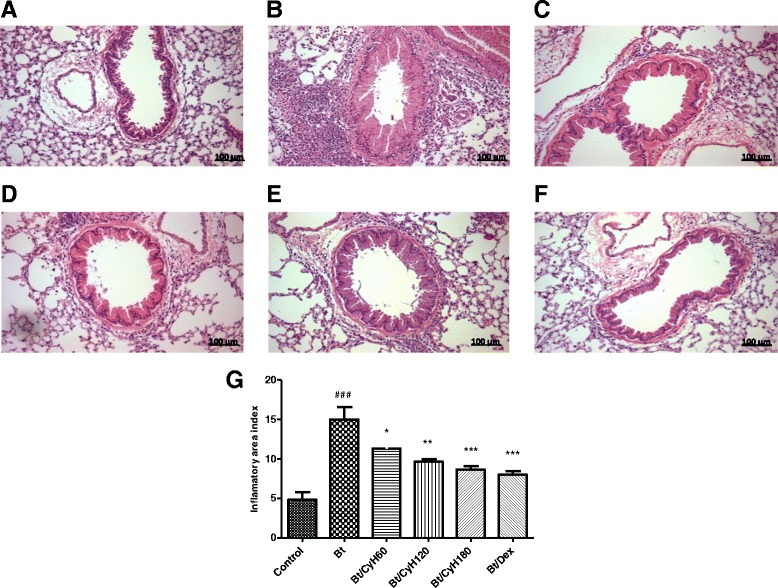
Effect of treatment with *Cy* on leukocyte infiltration in lung tissue of mice sensitized with *Bt* antigen. Sections were stained with hematoxylin-eosin (magnification × 400). Lung section from animal sensitized and treated with saline **a**, sensitized with *Blomia tropicalis* (100 μg/animal) and 4 mg/mL of [AL(OH)_3_] and treated with saline **b**, sensitized with *Bt* (100 μg/animal) and 4 mg/mL of [AL(OH)_3_] and treated with *Cy* hexane extract (CyH) at 60 mg/kg **c**, 120 mg/kg **d**, 180 mg/kg **e** or 3 mg/kg dexamethasone **f**. Quantification of lung inflammation **g**. Scale bar, 100 μm. Each column represents the mean of inflammatory area index of 5 mice, and the vertical bars represent the standard deviation of the mean. ### *p* < 0.001 vs. control; *p < 0.05; **p < 0.01; ***p < 0.001 vs. Bt group by ANOVA-Tukey

Figure 9b shows a cellular infiltrate that is characteristic of allergic asthma, including leukocyte infiltration around the bronchioles and perivascular region. Oral treatment with 60, 120 and 180 mg/kg *Cy* reduced inflammatory cell infiltration around the bronchioles (*p* < 0.05, Fig. 9c; *p* < 0.01, Fig. 9d and *p* < 0.001, Fig. 9e). As expected, treatment with 3 mg/kg Dex decreased inflammation in the lung tissue of Bt-immunized and challenged mice (*p* < 0.001, Fig. 9f). The reduction of inflammation was confirmed by quantification of inflammatory area.

### Treatment with *Cymbopogon citratus* hexane extract decreases mucus production in lungs of Bt-immunized animals

**Fig. 10:**
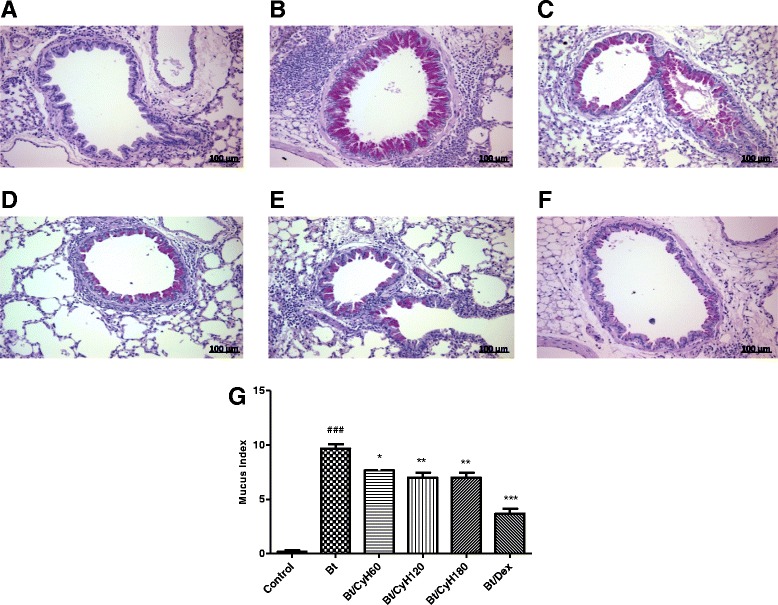
Effect of treatment with *Cy* on mucus production in lung tissue of mice sensitized with Bt antigen. Sections were stained with periodic acid-Schiff (magnification × 400). Sections were stained with hematoxylin-eosin (magnification × 400). Lung sections from animals sensitized and treated with saline **a**, sensitized with *Blomia tropicalis* (100 μg/animal) and 4 mg/mL of [AL(OH)_3_] and treated with saline **b**, sensitized with *Blomia tropicalis* (100 μg/animal) and 4 mg/mL of [AL(OH)_3_] and treated with *Cymbopogon citratus* hexane extract (CyH) at 60 mg/kg **c**, 120 mg/kg **d**, 180 mg/kg **e** or 3 mg/kg dexamethasone **f**. Scale bar, 100 μm. Each column represents the mean of the mucus indexes of 5 mice, and the vertical bars represent the standard deviation of the mean. ### *p* < 0.001 vs. control; *p < 0.05; **p < 0.01; ***p < 0.001 vs. Bt group by ANOVA-Tukey

In our experimental model of allergic asthma induced by mite *Blomia tropicalis*, intense mucus production can be observed in the lung tissue of Bt-sensitized and challenged animals (*p* < 0.001, Fig. 10b). In contrast, treatment with 60, 120 and 180 mg/kg hexane extract reduced mucus production (*p* < 0.05, Fig. 10c; *p* < 0.01, Fig. 10d-e). As expected, treatment with 3 mg/kg Dex decreased production of mucus in lung tissue of Bt-immunized and challenged mice (*p* < 0.001, Fig. 10f). The reduction of mucus production was further confirmed by quantification analysis as shown in Fig. 10g.

### Treatment with *Cymbopogon citratus* hexane extract decreases the expression of NF-κB p65 in the lungs of Bt-immunized animals

**Fig. 11:**
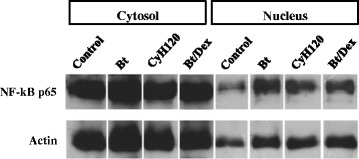
Detection of NF-κB expression in lung tissue of mice sensitized with *Bt* antigen. A/J mice were sensitized and treated with saline (Control), sensitized with *Blomia tropicalis* (100 μg/animal) and 4 mg/mL of [AL(OH)_3_] and treated with saline (Bt) or sensitized with *Blomia tropicalis* (100 μg/animal) and 4 mg/mL of [AL(OH)_3_] and treated with *Cymbopogon citratus* (Cy) hexane extract at 120 mg/kg (Bt/CyH120) or 3 mg/kg dexamethasone (Bt/Dex). Each lane is representative of the results obtained from five animals

**Fig. 12:**
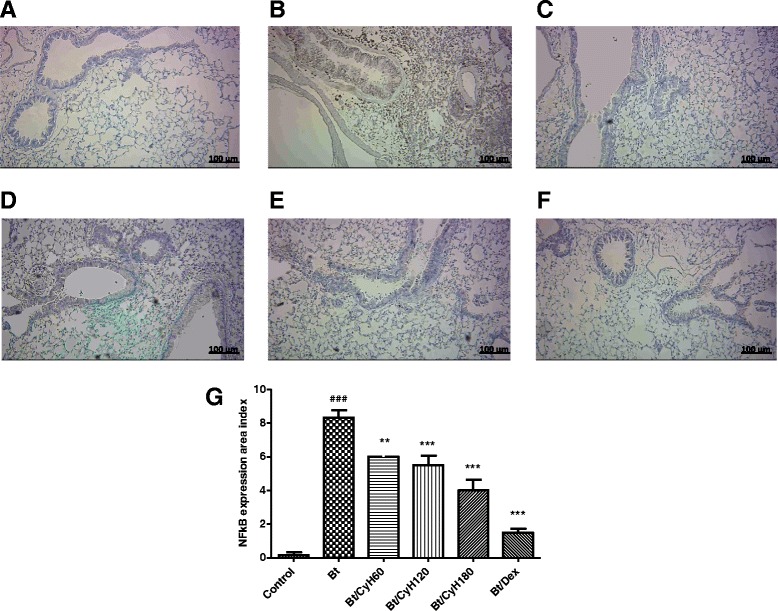
Effect of *Cymbopogon citratus* treatment on expression of NF-κB p65 in the lung tissue of mice sensitized with *Bt* antigen. Sections were stained by immunohistochemistry (magnification × 400). Lung sections from A/J mice sensitized and treated with saline **a**, sensitized with *Blomia tropicalis* (100 μg/animal) and 4 mg/mL of [AL(OH)_3_] and treated with saline **b**, sensitized with *Blomia tropicalis* (100 μg/animal) and 4 mg/mL of [AL(OH)_3_] and treated with *Cymbopogon citratus* hexane extract (CyH) at 60 mg/kg **c**, 120 mg/kg **d**, 180 mg/kg **e** or 3 mg/kg dexamethasone **f. g** presents the quantification of NF-κB p65 expression, Scale bar, 100 μm

Note from Fig. 11 the increased nuclear expression of NF-κB in Bt-immunized and challenged mice when compared to the control group. Treatment by oral administration of 120 mg/kg hexane extract reduced the expression of NF-κB/p65 compared to Bt-immunized and challenged mice. As expected, treatment with 3 mg/kg dexamethasone decreased the expression of NF-κB/p65 compared to Bt-immunized and challenged mice. Fig. 12b shows the nuclear staining of anti-NF-κB p65 in various cells around the bronchioles (*p* < 0.001, Fig. 8g). Treatment (v.o.) with 60, 120, and 180 mg/kg doses of *Cy* and dexamethasone 3 mg/Kg (Fig. 12c-e) were able to inhibit the expression of NF-κB p65 in the lungs of Bt-immunized and challenged animals (*p* < 0.01 and *p* < 0.001, Fig. 12g). The reduction of NF-κB/p65 was further confirmed by quantification of expression area as shown in Fig. 12g.

## Discussion

*Cymbopogon citratus* is commonly used in folk medicine to treat nervous and gastrointestinal disturbances and as an antispasmodic, analgesic, anti-inflammatory, anti-pyretic, diuretic and sedative [21]. Studies on the extracts from *Cy* leaves have demonstrated antioxidant, anti-microbial and anti-fungal activities [22]. The chromatogram obtained by gas chromatography coupled with mass spectrometry showed the main compounds with different profile for nonpolar substances from *Cy* extract may be attributed to the extraction method. Of the substances identified some have been previously observed in aqueous extract of *Cy*, such as β-eudesmol, that has anti-inflammatory activity by suppression of the NF-kappaB-expression [22, 23]. However, no previous study has explored the anti-allergic potential of *Cy*.

We have previously demonstrated the ability of natural products to modulate inflammatory cell recruitment to lung tissue [10]. Eosinophilia, eosinophil migration to the lungs, and their secreted products, such as inflammatory cytokines, are important factors that determine the severity and exacerbation of allergic diseases [18]. The present study was performed using a murine model of allergic disease induced by a common aeroallergen described by our group [9]. This work showed that treatment with *Cymbopogon citratus* reduced the infiltration of total leukocytes, particularly eosinophils, mucus production, and NF-κB expression in the lung tissue of Bt-sensitized animals. Treatment with the extract also reduced the production of eosinophil peroxidase, and suppressed levels of IgE, IgG1 and IL-4. Our results show that *Cy* suppressed EPO activity at all tested doses in Bt-sensitized animals. These data are related to the reduction in the number of eosinophils in the BAL of animals treated with *Cymbopogon citratus*, highlighting the important role of *Cy* extract in reducing inflammation and remodeling of lung tissue in allergic asthma. An emerging concept asserts that the nature and intensity of granulocytic infiltration of the airways, in particular the presence or absence of increased numbers of eosinophils, defines disease pathophysiologically and clinically [24].

The majority of asthma cases are associated with Th2-type T-lymphocyte–driven cell recruitment and mediator release involving mast cells, eosinophils, basophils, and macrophages that contribute to chronic, sub acute, and acute inflammatory responses [25]. Inflammatory cytokines such as IL-4, IL-5 and IL-13 are induced in the lung during asthma exacerbations [25]. To evaluate the mechanism of the *Cy*-mediated modulation of eosinophil infiltration, we investigated the effect of *Cymbopogon citratus* on the production of the Th2-type cytokine IL-4. Inhibition of inflammation is an essential process to establish homeostasis. We observed reduced levels of IL-4 after oral administration of 60 mg/kg, 120 mg/kg and 180 mg/kg *Cy* extract when compared to Bt-sensitized and treated with saline animals.

An important, consistent feature of asthma is the production of excess, altered mucus that blocks peripheral airways [25]. In asthma, there is ample evidence for goblet cell metaplasia involving the conducting airways; IL-4 and IL-13 also induce the production of TGF-β by epithelial cells that, through autocrine signaling, results in the mucus metaplasia that is characteristic of Th2-mediated inflammation [24]. Animals treated with the *Cy* hexane extract showed reduced mucus production in lung tissue, at all doses administered, stained with PAS. These results suggest decreased hyperplasia and hypersecretion of goblet cells in the airways obstruction a common feature in asthma pathology.

Our results showed that treatment with 180 mg/kg *Cy* extract decreased the levels of Bt-specific IgE in Bt-immunized mice; the levels of IgG1 were decreased when mice were treated with 120, 180 mg/kg *Cy* extract. Production of IgE depends on Th2-type cytokines, such as IL-4 [26]. Down-modulation of IgE production, as well as the reduction of IL-4, constitutes an important strategy for treating allergic diseases because the treatment of allergic asthma patients with IgE antibody was confirmed to attenuate eosinophilic airway inflammation [27] may have an important anti-allergic effect.

The molecular mechanisms underlying the protective effects of *Cy* in allergic asthma are not fully established. However, Edwards and colleagues (2009) found that the anti-inflammatory effects of *Cy* may be due to suppression of NF-κB activation [28]. One of the molecular mechanisms related to the effect of glucocorticoids is the inhibition of transcription factors such as NF-κB that are related to the production of inflammatory cytokines [29].

NF-κB exists in the cytoplasm in a latent form as a complex consisting of a dimer of DNA-binding subunits bound to an inhibitor, IκB. Activating agents, such as cytokines or lipopolysaccharide (LPS), induce the degradation of cytosolic IκB, promoting the release and nuclear translocation of NF-κB dimers. In most cells, the NF-κB is a heterodimer composed of the p65 and p50 subunits. This variant is the most potent gene transactivator among the NF-κB family and is the major NF-κB protein found in the nucleus of cytokine-stimulated cells. The transcription factor NF-κB is a central regulator of the transcriptional activation of a number of genes involved in proinflammatory responses [30].

Lee and colleagues (2008) found that Citral, a of the major compound of *Cy* inhibited iNOS expression, NO production and various LPS-induced pathways, including p38 mitogen-activated protein kinase (MAPK), c-jun NH2-terminal kinase (JNK) 1/2 and the transcription factor NF-kB [14]. In this work, we showed that treatment with our *Cy* extract at all administered doses reduced NF-κB/p65 expression in the lung tissue of Bt-sensitized animals, as determined by immunohistochemistry (Fig. 12). We also showed that treatment with *Cymbopogon citratus* at a dose of 120 mg/kg decreased NF-kB nuclear expression in lung tissue when compared to control groups of Bt-sensitized mice or mice sensitized with saline (Fig. 11). This may be one of the probable mechanisms mediating the anti-allergic effect of *Cy*. These findings are consistent with and corroborated by Francisco and collaborators (2011) which evidences the potential of *Cy* as source of compounds with anti-inflammatory properties, as well as the expression of NF-κB [21]. The data suggest that *Cymbopogon citratus* is a potential herb that can be used to control allergic asthma, reducing extensive infiltration of inflammatory cells into the lungs while also potentially inhibiting NF-κB expression in the lung. Further studies are needed to verify the exact mechanism of action of the *Cymbopogon citratus* extract, to identify the possible active compounds within the extract responsible for the properties observed and the mechanism whereby it occurs.

## Conclusion

The results presented in this study demonstrate the anti-allergic activity of *Cymbopogon citratus* extract in an experimental murine model of allergic asthma. Additional studies are being conducted in our group to describe possible mechanisms involved in allergic disease control by using *Cy*, as well as, isolated *Cy* compounds.

## Abbreviations

BAL: Bronchoalveolar lavage
BSA: Bovine serum albumin
Bt: *Blomia tropicalis*
ECL: Enhanced chemiluminescence
ELISA: Enzyme immunoassay test
EPO: Eosinophil peroxidase
HBSS: Hanks’ balanced salt solution
HCl: Hydrochloric acid
IL: Interleukin
IL-4: Interleukin 4
IL-5: Interleukin 5
IL-13: Interleukin 13
IFN-γ: Interferon gamma
i.n.: Intranasal
IgE: Immunoglobulin E-type
LPS: Lipopolysaccharide NaCl Sodium chloride
NaOH: Sodium hydroxide
NF-κB: Nuclear factor-κB
OVA: Ovalbumin
OD: Optical density
PAS: Periodic acid schiff
PBS: Phosphate-buffered saline
PBST: Phosphate-buffered saline containing 0.05 % Tween
PWM: Pokeweed mitogen
RPM: Revolutions per minute
s.c.: Subcutaneous
Th1: T helper lymphocyte type 2
Th2: T helper lymphocyte type 2
Treg: Regulatory T lymphocytes
v.o.: Oral
RIPA: Lysis buffer system
iNOS: Inducible nitric oxide synthase
